# A Generic Multi-Layer Architecture Based on ROS-JADE Integration for Autonomous Transport Vehicles

**DOI:** 10.3390/s19010069

**Published:** 2018-12-25

**Authors:** Jon Martin, Oskar Casquero, Brais Fortes, Marga Marcos

**Affiliations:** 1 Faculty of Electrical Engineering, Precision Engineering and Information Technology in the Technische Hochschule Nuernberg Georg Simon Ohm, 90489 Nuremberg, Germany; 2 Systems Engineering and Automatic Control Department, Faculty of Engineering in Bilbao, University of the Basque Country (UPV/EHU), 48013 Bilbao, Spain; oskar.casquero@ehu.eus (O.C.); brais.fortes@gmail.com (B.F.); marga.marcos@ehu.eus (M.M.)

**Keywords:** flexible manufacturing, transportation systems, ATV, AGV, robotic framework, multi-agent systems, ROS, JADE

## Abstract

The design and operation of manufacturing systems is evolving to adapt to different challenges. One of the most important is the reconfiguration of the manufacturing process in response to context changes (e.g., faulty equipment or urgent orders, among others). In this sense, the Autonomous Transport Vehicle (ATV) plays a key role in building more flexible and decentralized manufacturing systems. Nowadays, robotic frameworks (RFs) are used for developing robotic systems such as ATVs, but they focus on the control of the robotic system itself. However, social abilities are required for performing intelligent interaction (peer-to-peer negotiation and decision-making) among the different and heterogeneous Cyber Physical Production Systems (such as machines, transport systems and other equipment present in the factory) to achieve manufacturing reconfiguration. This work contributes a generic multi-layer architecture that integrates a RF with a Multi-Agent System (MAS) to provide social abilities to ATVs. This architecture has been implemented on ROS and JADE, the most widespread RF and MAS framework, respectively. We believe this to be the first work that addresses the intelligent interaction of transportation systems for flexible manufacturing environments in a holistic form.

## 1. Introduction

Different international roadmaps [1,2,3] for the digitalization of manufacturing systems deal with the need of new networked technologies that help factories adapt to current market demands, namely: on-demand production, shorter lifecycles, mass-customization schemes, high quality standards, rising speed of delivery and yet lower fixed costs. To meet those demands, flexibility, adaptability and reactivity have become the main characteristics of modern manufacturing systems: flexibility to achieve product customization without redundant production lines; adaptability for an easy and economic reconfiguration of the production processes; and reactivity against disturbances, such as failures and last-minute changes. These characteristics, in turn, lead to two design principles of the factory of the future: (a) connectivity, consisting on the ability of different manufacturing entities (machines, robots, warehouses, operators, etc.) to communicate with each other; and (b) decentralized decisions, consisting on the self-organization of manufacturing entities that negotiate among them to perform tasks and resolve conflicts. Therefore, each manufacturing entity in the factory of the future can be represented as an individual Cyber Physical Production System (CPPS) in the need of social abilities to achieve its function, either autonomously or cooperatively [1].

The Autonomous Transport Vehicle (ATV) is a robotic CPPS that plays a key role in building such intelligent manufacturing. Replacing the traditional fixed conveyor belts with flexible transportation robots and modular intelligent machines that manage their own material handling enables a quick and cost adequate reconfiguration of the production system (flexibility and adaptability) [4,5]. Furthermore, production orders do not need to follow a determined assembly sequence since the ATVs can perform unplanned, on demand deliveries (reactivity), e.g., to bring an unfinished product from a broken machine to another one that has some free operation time on its schedule. Research on autonomous transportation has been mainly focused on the design of robust software algorithms, libraries and tools, and on the integration of heterogeneous hardware components [6]. These works should be complemented in order to add social abilities to achieve efficient transportation tasks in changing environments. This can be summarized in two main requirements (MR):

- MR1: An ATV needs to interact with other ATVs to perform complex transportation tasks or resolve conflicts.
- MR2: An ATV needs to interact with other heterogeneous, non-robotic CPPSs in the factory environment to offer or require services, e.g., to offer transportation services to machines or to require a free charging station when its battery status reaches a critical level.

As commented above, the development of ATVs, being robotic devices, can be achieved using a Robotic Framework (RF), as it provides hardware abstraction and software components for the creation of complex and robust robot behaviors in diverse applications and across a wide variety of robotic platforms. Nevertheless, RFs—such as the popular Robot Operation System (ROS)—have been focused on developing single robot functionalities and have not included social abilities within their inherent characteristics. There are, however, specific RF packages—such as *multimaster_fkie* of ROS—that allow the development of distributed robotic systems at the expense of introducing latencies that can be intolerable [7]. RFs must now evolve aiming at improving social abilities among robots (MR1) and allowing the interaction of robots with non-robotic entities (MR2).

To overcome the lack of social abilities inherent to RFs, [8] proposed the combination of RF with already proven and reliable Multi-Agent Systems (MAS) to create the so-called Multi-Agent Robotic Systems (MARS). MAS are designed to manage distributed and changing environments where intelligent and loosely-coupled software components (agents) that made up the system do have to interact with each other to perform tasks [9,10,11,12]. MAS technology has been proved as a natural way to meet the socialization requirements among different manufacturing entities in many industrial domains [13]. In fact, the concept of Industrial Agent is related to the implementation of CPPSs as agents [14,15]. Thus, the application of the MAS paradigm to RFs contributes to the *agentification* of an ATV, a process by which an ATV would become an agent that can socialize with other agents in the factory.

In this context, this work contributes to the definition of a generic multi-layer architecture for enabling the MARS social abilities in ATVs and, thus, fulfilling MR1 and MR2. It integrates ROS and JADE, both open-source and widely spread RF and MAS framework, respectively. It consists of a customizable service-based architecture that uses distributed decision-making. A set of architecture components have been defined that, being generic, can be customized and replicated to meet the application requirements. The higher layer main goal is to offer the services of the ROS entity to other agents in the environment. The intermediate layers are designed for efficient execution, while the lowest layer constitutes the inner control of the robotic device. The architecture has been validated in the field of flexible manufacturing [16]. A MAS-based middleware has been developed aiming at supporting production reconfiguration. There, the ROS-JADE integration architecture is used to implement the ATV agent that offers transportation and other ATV rescue services, and demands charging services from charging stations.

The rest of the paper is organized as follows: Section 2 summarizes the related work; Section 3 describes the ROS-JADE integration architecture; in Section 4, three uses cases are presented to demonstrate how the architecture can accomplish significant goals in some meaningful scenarios; finally, Section 5 collects the conclusions of the work.

## 2. Related Work

This section comprises some research work dealing with the key aspects of this paper, i.e., (a) the importance of an ATV for achieving flexibility in manufacturing, (b) the efforts to create multi-robot architectures using MAS, and (c) the use of MAS in manufacturing for integrating the ATV with other CPPSs in heterogeneous and distributed environments.

One possible solution for achieving high-grade of production flexibility and adaptability at plant level is to distribute the factory into modular manufacturing entities capable of talking to each other and to other CPPSs, and deciding production reconfiguration in case of context changes. In this case, production orders do not need to follow a determined assembly sequence, since the sequence could be defined by the manufacturer itself or by the current machine-resources availability [17]. There are many works that endorse the idea of using Autonomous Transport/Ground Vehicles for managing the transportation logistics. For instance, [5] presents the Kiva system, a real application for autonomous warehouse item storage-and-delivery where thousands of robots work in human-free environmental areas performing transportation orders as a part of the Amazon Robotics logistics fleet. However, the task allocation is done in a centralized way [18]. Other works in the context of autonomous material handling systems for modular manufacturing processes are [4,19]. Reference [19] presents a plug-and-play mobile conveyor module called KARIS that is distinguished by building adaptable robot cooperative teams and addressing the decentralization problem for intra-logistics task in [20]. On the other hand, the Multishuttle Move [4] presents a fusion of conventional shuttle and automated guided vehicle. Reference [21] deals with the decentralization issue for path planning and navigation by means algorithms for coordinating ATVs.

As commented above, ROS multimaster was a work towards allowing ROS based multi-robot systems to communicate in a distributed ay, but it introduces unaffordable communication latencies. Currently, to overcome this and other limitations (e.g., improving network communications) ROS2 [22] is being developed. However, the communication is yet limited to ROS-based systems and an additional communication channel is needed to communicate with other heterogeneous CPPSs.

On the other hand, many research works take advantage of the MAS paradigm to build intelligent multi-robot systems. For example, [23] presents a group of cooperating robots, where an intelligent software agent “moves” from one physical platform to another (e.g., due to a low battery event in the current platform) by using the agent mobility capabilities. Other works like [24] make use of a service oriented multi-agent platform [25] for the analysis, design and implementation of complex systems where the data sources and data processing are distributed. Unfortunately, most of these systems present ad-hoc solutions for specific applications and cannot be easily customized because of the use of non-standard frameworks. References [26,27] use the framework JADE to build direct collaboration between industrial robots and humans.

There is an increasing interest on building generic multi-layer architectures for multi-robot collaboration based on the integration of RF and MAS. These works focus on developing intelligent multi-agent networks composed by robots and smart sensors in service applications. AutoRobot [28,29] combines ROS and JADE to enable support to autonomous and rational service robots. It offers a series of reusable packages, templates and tools to help with the integration of new agents (robot or sensor) within their framework. However, it mainly focuses on single robot requirements, relegating the socialization issues for future work. Reference [30] proposes an architecture to control and coordinate team working robots. However, the coordination among agents occurs over a central supervisor component which detects and avoids collision conflicts. Reference [31] is another three-layered architecture for enabling service robotics in intelligent environments. Specifically, they present the interaction of a mobile robot with smart light and door agents. Here, a centralized component acts both as a global knowledge container and as a central coordinator of other system components. In summary, despite the noticeable efforts on the research of RF-MAS integration architectures, the decentralization issue remains unsolved, limiting one of the main benefits of using MAS.

Finally, MAS technology has also been proposed to achieve flexible manufacturing. ADACOR [32] and PROSA [33], represent two of the most referenced projects in the industry domain that integrate MAS frameworks into holonic manufacturing systems. Both works provide a catalog of *agentified* CPPSs that simplifies the developments of agent-based control systems. Nevertheless, these works have not deal with the problematic of multi-robot systems and it is therefore necessary to develop a generic RF-MAS architecture to easily integrate heterogeneous groups of ATVs in such a multi-agent system.

## 3. Generic ROS-JADE Integration Architecture

This section presents the generic multi-layered architecture. Initially, the specific requirements to be met by the architecture are identified. Then, every layer of the architecture, its main goal and the key generic components are described in detail, highlighting how they can be customized and replicated.

### 3.1. Architecture Requirements

While general requirements related to multi-robot systems have already been referred in the existing literature [8,34,35], the specific requirements related to the socialization of ATVs with their environment in a flexible manufacturing process are yet to be defined. From MR1 and MR2, we identify a list of more specific requirements (SR) related to the ATV and its socialization abilities (Table 1).

With regard to SR1, the ATV services must be published and made available to other agents. Negotiating capabilities are required to reach agreements with other ATVs during task allocation process. SR2 comes from the need of being ready to socialize, while abstracting this duty from low-level, robot-dependent functional tasks. As for SR3, event management mechanisms are needed to notify ATV state changes that may affect other CPPSs in the manufacturing environment, e.g., if its battery level is low, the ATV must stop offering services until recharged. Regarding SR4, the ATV functionality must be adaptable to context changes, e.g., it must reduce the maximum navigation speed in presence of human operators or while navigating in restricted areas. Finally, SR5 indicates the ATV must be capable of interacting not only with other ATVs, but also with other heterogeneous, non-robotic CPPSs in the factory, e.g., machines demanding transportation services. The following sub-sections detail the architecture and how the specific requirements are met.

### 3.2. Multi-Layer Architecture

**Figure 1:**
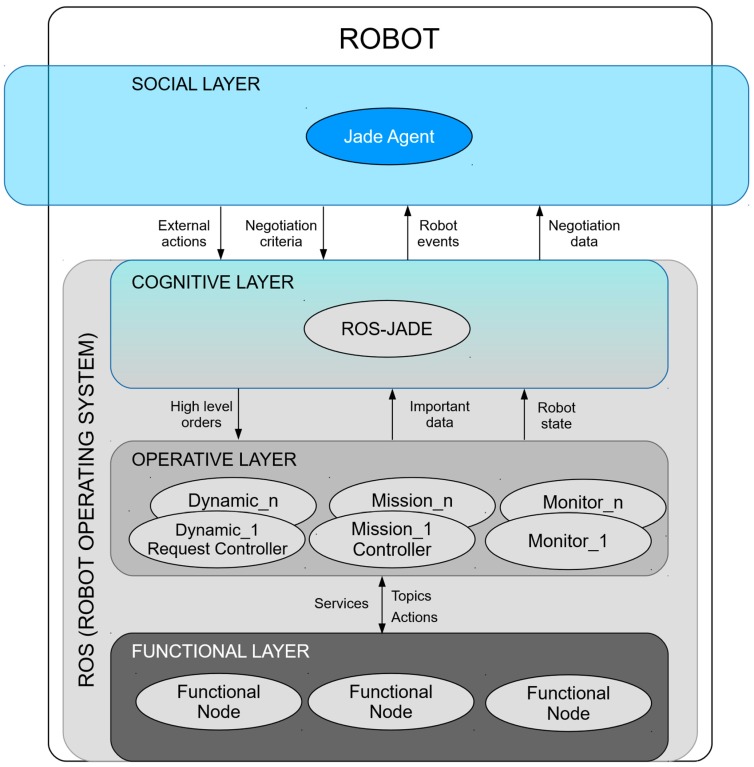
ROS-JADE integration multi-layer architecture.

The 4-layer ROS-JADE integration architecture is illustrated in Figure 1. Each layer contributes to meet at least one of the requirements in Table 1. The upper layer (social) is responsible for the interaction with other CPPSs, contributing to fulfill SR1 and SR5. The lower layer (functional) deals with the basic ATV control (sensors, actuators, robotic algorithms...) and is implemented by means of a RF. The intermediate layers are responsible for abstracting the social behavior from the ATV functionality and, at the same time, they are in charge of pre-processing and storing the information needed at the social layer for achieving fast negotiation response. Besides, these layers are also in charge of transmitting information and events between the functional and social layers. Namely, the cognitive layer performs the actual integration of RF and MAS frameworks, performing the bi-directional communication between the social layer and the operative layer. To fulfill SR2, the operative layer pre-processes and the cognitive layer stores the information that the social layer might need. The operative layer also allows responding to events coming from the social layers through online tuning of ATV parameters (SR4) as well as transmitting events coming from the functional layer to the social one (SR3). The following subsections describe each layer in greater detail, emphasizing the different type of generic components at every layer, which can be replicated to meet application requirements, and their main characteristics.

#### 3.2.1. Social Layer

The social layer consists of one MAS Agent (in the prototype, a JADE Agent; hereafter, the Agent) that offers at least transportation services (SR1) and communicates with the rest of the CPPSs in the manufacturing system, negotiating and cooperating with them (SR5). However, since a robotic system is made up of multiple components, it is possible to offer additional services, such as the use of integrated cameras for monitoring purposes or localization services, giving the possibility of global localization to other CPPSs having limited resources.

The Agent represents the global intelligence of the ATV in the system. Its main functions are offering ATV capabilities as services, dealing with the requests of other agents in the system, and negotiating with other ATVs to decide which ATV will actually perform the service. Whenever the Agent gains a negotiation or receives an event from other CPPS, the social layer transmits the order downwards through utilities of the cognitive layer, making it independent from RF and ATV functionalities. The Agent may also request services itself, e.g., when due to a failure the ATV needs to be rescued.

During the initialization phase, the Agent registers its transportation services (and others if necessary) in the Directory File (DF) of the MAS platform. The DF is also known as the Yellow Pages service and acts as a directory of the existing resources. Other system agents in the manufacturing environment can consult this directory and look for available ATVs that offer certain services. Similarly, if agents corresponding to charging stations have also registered their services, ATVs with low battery ask the DF for available charging services. The negotiation and service allocation through distributed decision making will be described in the use cases (Section 4).

#### 3.2.2. Cognitive Layer 

**Figure 2:**
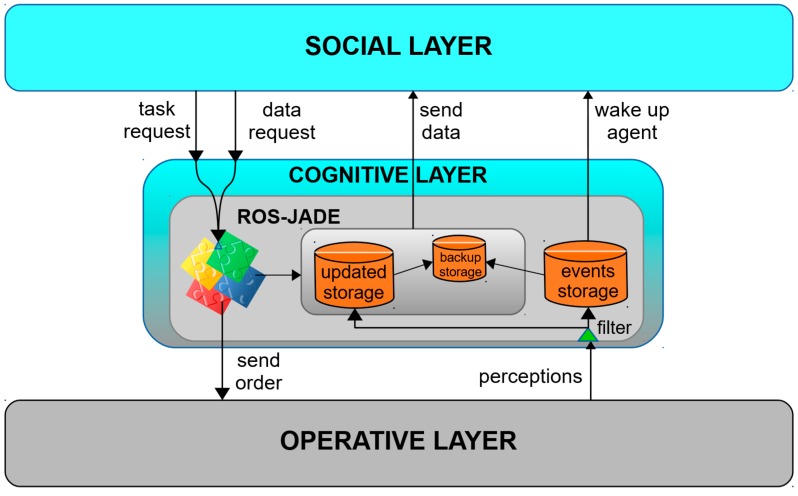
ROS-JADE component (cognitive layer).

This layer performs the actual integration of the RF and MAS frameworks. It consists of a unique component (the ROS-JADE node) whose main function is communicating the agent at the social layer with the operative layer. This includes sending information in both directions, transmitting events coming from the lower layer and transmitting orders from the social layer. Besides, and very important to fulfill the fast reaction specific requirement (SR2), it stores different types of pre-processed and updated information that the agent at the social layer might need for negotiation. Figure 2 illustrates the generic data structure that is implemented at this layer.

- *Updated storage*. It stores all the information that is completely necessary for negotiation or for the social relationship between agents. In this way, the last information received from each type of data is stored, keeping the database updated and waiting for the Agent requests.
- *Event storage*. All events that need immediate management by the Agent are stored in this separate data table. This allows the cognitive layer awakening the Agent upon event reception.
- *Backup storage*. It keeps a record of all the information that has been stored for a certain period of time. These data can be used for recovery purposes in case of a failure, or for learning or forecasting purposes for later analysis.
- *Low-level messages filter*. Before the information is stored in the report or event storages, the received perception messages are verified to be relevant for the managing high-level service. If not, they are ignored.

#### 3.2.3. Operative Layer

This layer has three main functions. First, it interprets high-level orders coming from the upper layers, generating the sequence of orders to be executed by the ROS nodes at the functional layer. This is the function of Mission Controller nodes. Second, it contributes to achieve an efficient response to service requests (SR2) by means of Monitor nodes which pre-process information that the social layer might need. Monitors also transmit internal events from the functional to the upper layers (SR3). Finally, Dynamic Request Controller nodes allow adaptation of ATV configuration parameters due to context changes, thus, contributing to meet SR4. It is important to remark that despite being strongly linked to the services offered by the ATV at the social layer, these nodes are kept simple to promote their reuse in different applications. In the following subsections, the three main components are described in detail.

(A) Mission Controller

**Figure 3:**
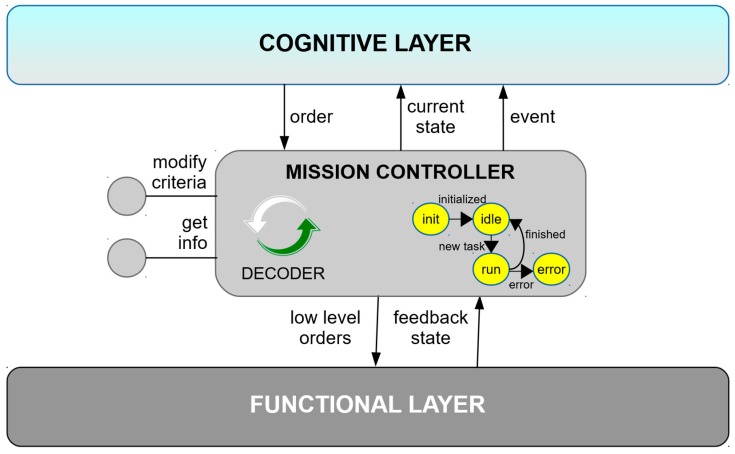
Mission Controller component (operative layer).

Mission Controllers are responsible for managing the execution of services, transforming orders into understandable subtasks for the ROS nodes at the functional layer, and managing them until their completion. Mission Controllers execute the state machine of Figure 3 and transmit to the upper layer the execution state or unexpected events preventing the service to be completed. In addition, these nodes offer services to modify their operating criteria or get information on demand.

(B) Monitors

**Figure 4:**
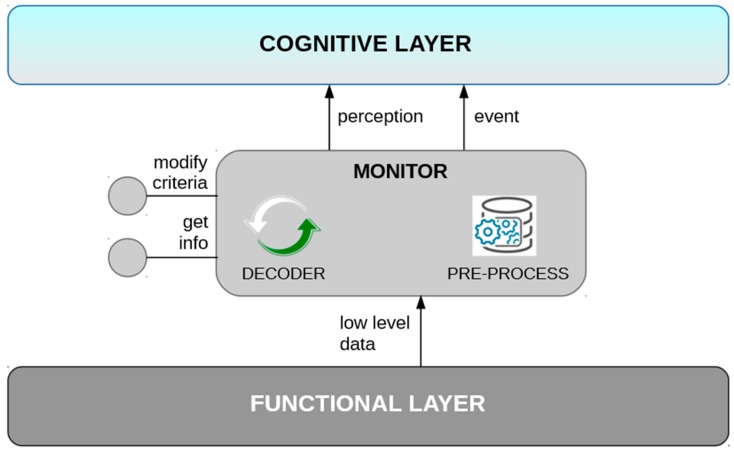
Monitor component (operative layer).

Monitors (Figure 4) are in charge of performing perceptions by means of pre-processing data read from the functional layer. Only relevant information changes (robot pose, battery status...) are transmitted to the upper layers, avoiding the saturation of communications due to high processing rates. The thresholds and criteria for pre-processing and publishing the information can be modified through the services that implement these nodes. It also allows reading on demand the information they process.

(C) Dynamic Request Controller

**Figure 5:**
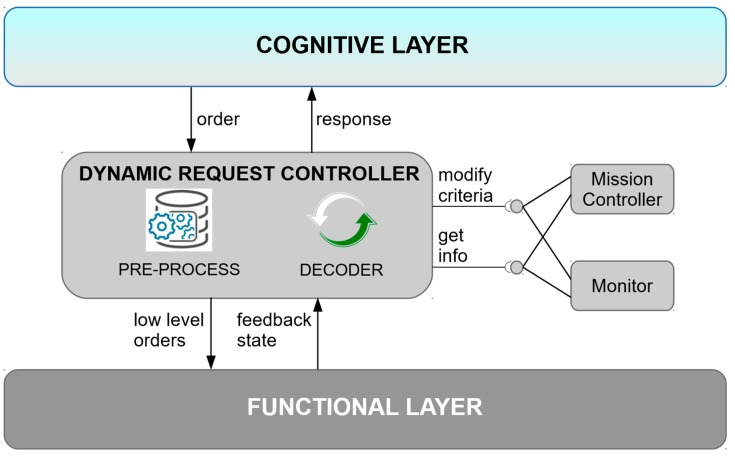
Dynamic Request Controller component (operative layer).

The objective of this type of node (Figure 5) is twofold. On the one hand, it is responsible for attending special requests of the social layer that affect Mission Controllers and Monitors. Two types of special requests can be distinguished: (a) return information, for which it uses the *get info* operation; or (b) modify parameters, for which it uses *modify criteria* operation. These requests can be used, e.g., to change the pose refreshing rate or to slow down the ATV when cooperating with other robots, respectively. On the other hand, Dynamic Request Controllers handle special situations where they make punctual requests to functional nodes (low level orders) and receive an instant response in return (feedback state); e.g., they can request, if necessary, to compute the path between two points.

In a normal processing situation, the ATV does not use the Dynamic Request Controller for any management. However, they permit dealing with particular requests from social agents that no other component of this layer is capable of addressing, reducing other nodes complexity and simplifying the architecture.

#### 3.2.4. Functional Layer 

This layer is made up by the RF components (ROS nodes) involved in the basic control functionalities of an ATV. Thus, these nodes manage the sensor and actuator components, and contain the basic algorithms to control the robot. A benefit of using ROS on this level is the availability of a huge variety of robotic component drivers (robot platforms, cameras, lasers, manipulators) and algorithms (for navigation, localization, perception, manipulation) tasks that can be customized as needed.

**Figure 6:**
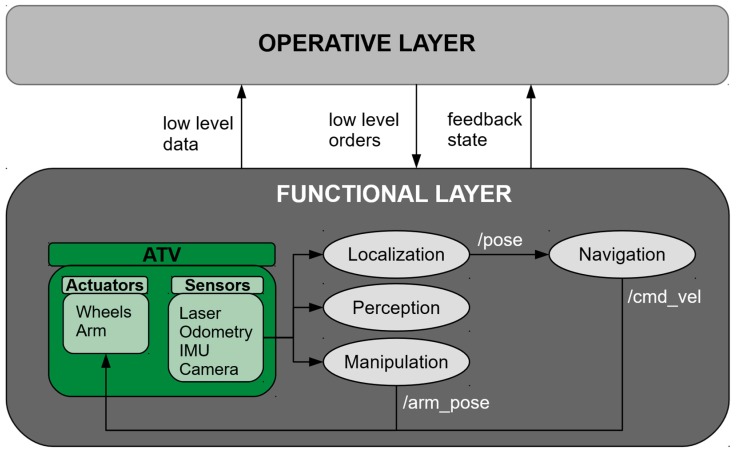
Functional layer.

As seen in Figure 6, an ATV will at least be composed of a mobile platform with differential or omni-directional wheels for the navigation; a localization system usually composed of the wheels-odometry combined with an IMU and a laser/camera/funk based localization system; perception components to detect obstacles or other desired features in the environment; a manipulation system such as an arm or a lift platform to manipulate/carry the load; and a computer that manages the control and organization of the different modules.

## 4. Use Cases

In the future, robots could autonomously manage the factory transportation from the moment raw material arrives until the final product is delivered. For that purpose, the ATV offers transportation services that could be used by machines to (a) get new raw material replenishment, (b) transport their operated sub-products to the next machine, or once finished, (c) deliver the finished product to the warehouse.

Apart from machines, an ATV should interact (a) with other CPPSs in the factory, such as other ATVs or charging stations along the shop-floor. These entities are represented as agents that socialize among each other to cooperate or compete, and thus, to reach their global goals. Moreover, the ATV could also receive notifications from smart sensors that inform about traffic events (traffic lights like) or the entering into a human-working area.

This section presents several use cases showing the socialization of ATVs among themselves and with other non-robotic CPPSs to demonstrate the benefits of combining RF and MAS. We will first show an ATV—Transportation Agent (TA)—noticing internal low battery levels and its interaction with available charging stations—Charging Station Agents (CSA). Secondly, a material replenishment order triggered by a machine—Machine Agent (MA)—and the resulting task allocation between available ATVs are shown. In the third case, a smart sensor informs the robot of entering a human-working area, triggering a robot reconfiguration. These agents register their services on the DF of the MAS platform.

**Figure 7:**
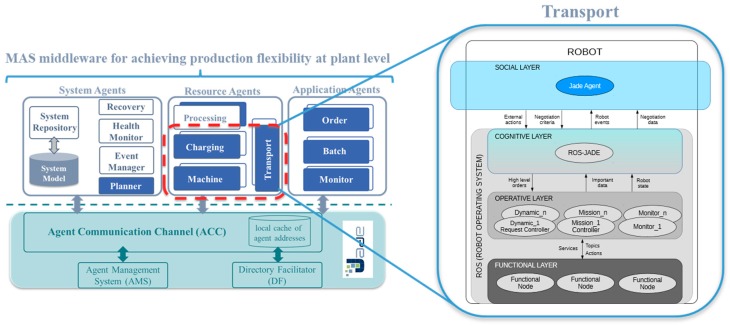
The architecture of the MAS middleware for achieving production flexibility at plant level and its relation to the ROS-JADE integration architecture.

**Figure 8:**
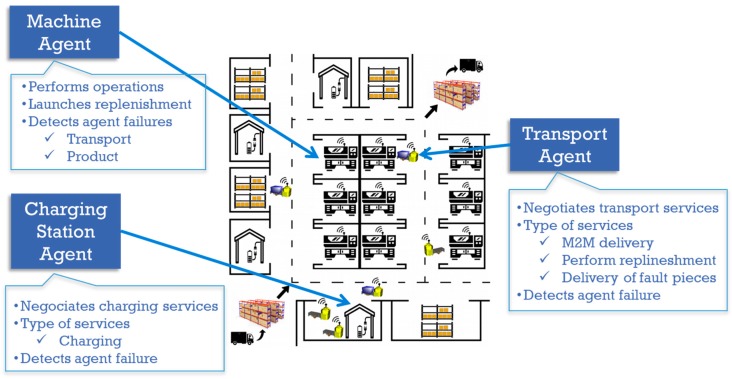
Factory layout for the validation of the uses cases.

The uses cases were validated from a functional point of view in a prototype of a MAS middleware for achieving production flexibility at plant level. Figure 7 shows the agent architecture of the middleware as well as how the ROS-JADE integration architecture is implemented in the TA. Figure 8 illustrates the factory layout of the validation made up of ATVs, charging stations and machines. ATVs are Kobuki robots running the TA, whereas machines and charging station agents are simulated in computers running their corresponding agent behaviors (MA and CSA, respectively).

### 4.1. Management of ATV Battery Health/Moving to a Waiting Place after Finishing a Task

**Figure 9:**
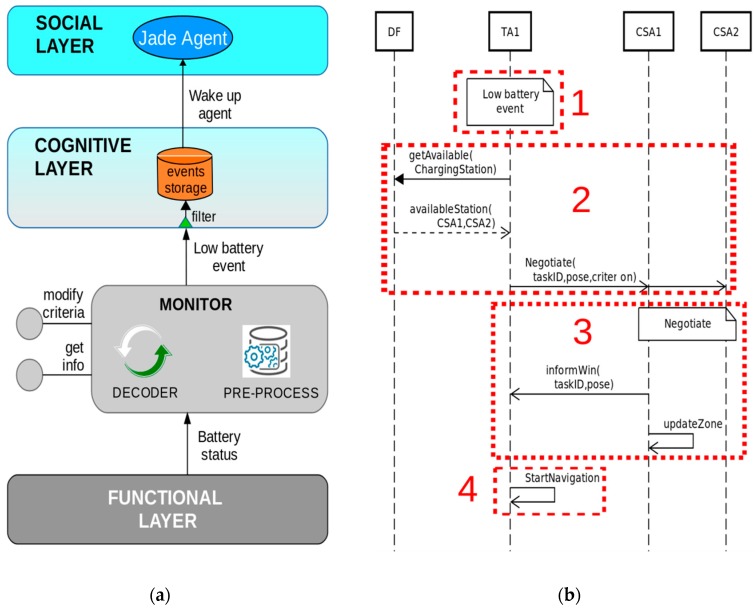
(**a**) Low battery event occurred in the ATV functional nodes and spread over the monitor and event storage components up to the social layer. (**b**) Sequence diagram representing a charging station service request by TA1.

When the ATV notices critical battery levels, it requires a free position in a charging station. The process is illustrated in Figure 9b and carried out in four steps: (1) The ATV agent (the TA) receives a low battery event issued from the functional layer (Figure 9a); (2) TA requests available power stations to the DF, triggering a negotiation among them; (3) CSAs negotiate under the specified criterion, for instance, nearest to the ATV, calculating their cost and sending it to other participants. The winner of the negotiation informs TA about the pose; (4) the ATV navigates to that pose.

When an ATV finishes a transportation task, a similar sequence as the represented in Figure 9b is initiated, as the ATV must leave the surroundings of the machine and move to a charging station.

### 4.2. Machine Material Replenishment

**Figure 10:**
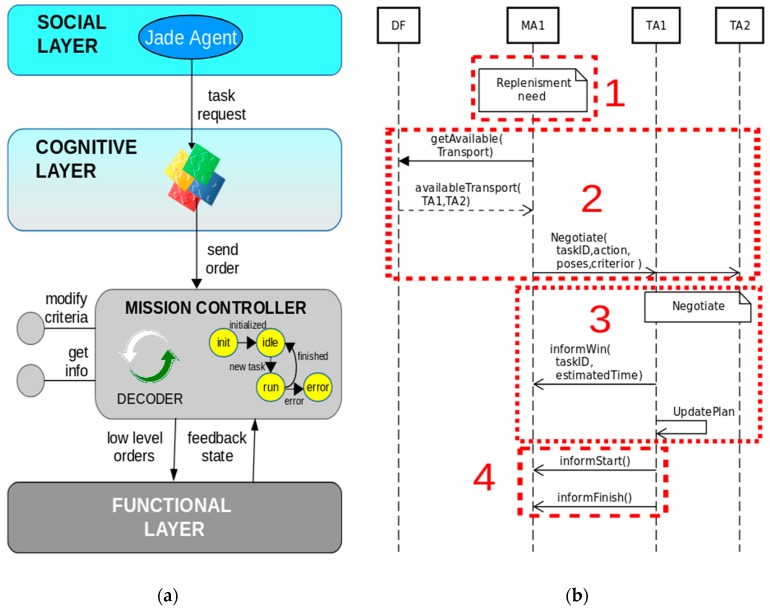
(**a**) New replenishment task request issued by the TA to the mission controller; (**b**) Sequence diagram representing a material replenishment service request by MA1.

The intelligent ATV provides high flexibility to manufacturing processes managing a dynamic plan to serve on-demand requests. This allows solving expected or unexpected events in the plant in an agile way. For instance, when a machine needs material to perform its operations, its agent (the MA) makes use of transport services to perform a material replenishment. Figure 10b presents the four steps to carry out a replenishment transportation service request: (1) The MA detects lack of material; (2) MA requests available transportation services and initiates a negotiation among TAs under a specified criterion; (3) The winner ATV notifies the MA; (4) The ATV starts the transportation task informing the MA about the beginning and the end of the task. Figure 10a shows the fourth step performed by the TA winner which requests the transportation task to the mission controller.

### 4.3. ATV Speed Adaptation on Demand

There are manufacturing entities that are not intelligent enough to be represented as agents, but that could still transmit important information to ATVs, triggering, if necessary, ATV reconfiguration. This is the case of restricted area sensors. They can be easily settled in strategic locations (by Beacons, WiFi or other desired technology) to alert about the entering into a special area. In order to receive this information, the robot must include the hardware component that receives the sensor signal in the functional layer and implement a new monitor in the operational layer to pre-process this information and create the events that wake up the social agent (similarly as in Figure 9a). These events will trigger then the ATV reconfiguration.

**Figure 11:**
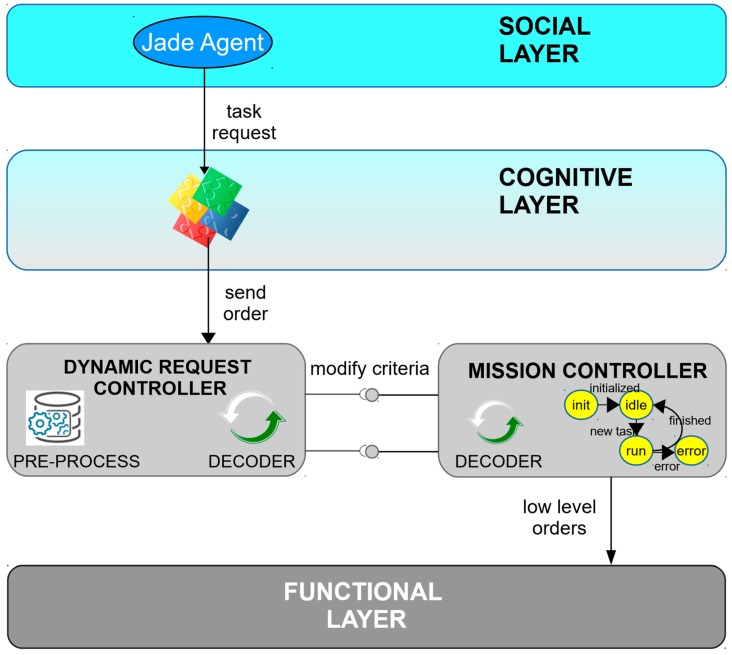
ATV reconfiguration triggered by the Dynamic Request Controller to increase the ATV velocity.

For example, a robot without load could navigate at maximum speed within an automated warehouse where human operators are not allowed to enter. Thus, the transportation performance is improved, while the risk of hurting humans is avoided. As reconfiguration actions are not directly related to the transportation task, functional nodes do not manage them, this is a typical function of Dynamic Request Controllers, which in turn set new navigation criteria (in this case, new speed) for the mission controller (see Figure 11).

## 5. Conclusions and Future Work

The ATV has been pointed as one of the key enablers of modern manufacturing, providing the necessary flexibility, adaptability and reactivity to the transportations systems of the factory of the future. However, to perform their transportation tasks, ATVs will not only have to interact among them, but also with other CPPSs and with their environment in an intelligent way.

As far as authors know, this is first work that addresses intelligent interaction of transportation systems in flexible manufacturing environments using a holistic approach, adding social abilities to an ATV and transforming it into an Intelligent Transportation Vehicle (ITV). The core set of specific requirements related to the socialization of ATVs have been identified. This work contributes a generic multi-layered architecture proposal that integrates a RF, responsible for the control of the main robotic functionalities of the ATV, with a MAS framework that provides the required social abilities. The layered architecture meets the requirements while abstracting the social abilities from the control functionalities, decoupling attention to service requests from the high frequency information refreshing at functional level, promoting control code re-use and separation of concerns, as higher-level services can be adapted without modifying the functionality and vice versa. The division on layers is done with efficiency and modularity in mind, avoiding functionality overlapping between layers. This architecture has been implemented on ROS and JADE, the most widespread RF and MAS framework, respectively, which offer the necessary base to develop a MARS. In addition, the uses cases presented in this work contribute to illustrate how ATVs based on this architecture are able to collaborate with machines, charging stations or environmental sensors in the factory, efficiently responding to transportation service requests and adapting to context changes.

Further work within this line of research should include the validation of the ROS-JADE integration using real settings and assessing the efficiency of the architecture in real environments having a big number of agents. Besides, despite this work has been focused on the transportation systems, the architecture can be easily implemented in any other application field that needs from endowing intelligence and sociability to robotic systems. In fact, the architecture permits to have more than a single agent, depending on the complexity of the application, the robotic component involved, and the processing unit that manages the robotic services. Thus, given that the modularity and generalization of the different layers and their components make them independent from the chosen RF and MAS framework, the architecture should be validated in other frameworks different from ROS and JADE.

## Figures and Tables

**Table 1:** Specific requirements (SR) of an ATV in a flexible manufacturing process.

Identifier	Brief Description
SR1	Offer transportation services in competition or collaboration with other ATVs.
SR2	Give efficient responses to service requests.
SR3	Notify significant transportation events at a social level.
SR4	Allow reactivity through online service tuning.
SR5	Communicate with any type of CPPS.
